# Spatio-temporal clustering analysis and its determinants of hand, foot and mouth disease in Hunan, China, 2009–2015

**DOI:** 10.1186/s12879-017-2742-9

**Published:** 2017-09-25

**Authors:** Xinrui Wu, Shixiong Hu, Abuaku Benjamin Kwaku, Qi Li, Kaiwei Luo, Ying Zhou, Hongzhuan Tan

**Affiliations:** 1 Department of Epidemiology and Health Statistics, Xiangya School of Public Health, Central South University, Changsha, Hunan 410008 People’s Republic of China; 2 Hunan Center for Disease Control and Prevention, Changsha, Hunan 410078 People’s Republic of China; 3 Department of Epidemiology, Noguchi Memorial Institute for Medical Research, College of Health Sciences, University of Ghana, PO Box LG581, Legon Accra, Ghana

**Keywords:** HFMD, Spatial autocorrelation, Spatio-temporal analysis, Tendency analysis, Autologistic regression model, Determinants

## Abstract

**Background:**

Hand, foot and mouth disease (HFMD) is one of the highest reported infectious diseases with several outbreaks across the world. This study aimed at describing epidemiological characteristics, investigating spatio-temporal clustering changes, and identifying determinant factors in different clustering areas of HFMD.

**Methods:**

Descriptive statistics was used to evaluate the epidemic characteristics of HFMD from 2009 to 2015. Spatial autocorrelation and spatio-temporal cluster analysis were used to explore the spatial temporal patterns. An autologistic regression model was employed to explore determinants of HFMD clustering.

**Results:**

The incidence rates of HFMD ranged from 54.31/10 million to 318.06/10 million between 2009 and 2015 in Hunan. Cases were mainly prevalent in children aged 5 years and even younger, with an average male-to-female sex ratio of 1.66, and two epidemic periods in each year. Clustering areas gathered in the northern regions in 2009 and in the central regions from 2010 to 2012. They moved to central-southern regions in 2013 and 2014 and central-western regions in 2015. The significant risk factors of HFMD clusters were rainfall (OR = 2.187), temperature (OR = 4.329) and humidity (OR = 2.070). The protect factor was wind speed (OR = 0.258).

**Conclusions:**

The HFMD incidence from 2009 to 2015 in Hunan showed a new spatiotemporal clustering tendency, with the shifting trend of clustering areas toward south and west. Meteorological factors showed a strong association with HFMD clustering, which may assist in predicting future spatial-temporal clusters.

**Electronic supplementary material:**

The online version of this article (10.1186/s12879-017-2742-9) contains supplementary material, which is available to authorized users.

## Background

Hand, foot and mouth disease (HFMD) is a highly contagious disease caused mainly by human enterovirus 71 (EV71) and coxsackievirus A16 (CoxA16), [1]. Although the infection is typically mild and self-limiting, infants and children, especially those under 5 years old are at greatest risk of severe disease affecting the central nervous system [2]. Currently, there are no available vaccines, chemoprophylaxis and effective anti-virus therapies for dealing with HFMD [3, 4].

The past decades have witnessed several outbreaks of HFMD across the world, affecting millions of people in countries such as Bulgaria, Australia, Japan, and Vietnam [5–8]. In March 2008, a nation-wide epidemic of HFMD started in Fuyang City, Anhui Province, China, recording 6049 cases and 22 deaths [9]. In view of these influences, HFMD has been listed as a reportable disease by the Chinese Ministry of Health to facilitate better disease prevention and control. Surveillance data shows that over 7.2 million cases and 2457 fatal cases were reported in China between 2009 and 2012, making HFMD the top reportable disease in China during the period under review [10].

Many studies showed that some climatic factors were associated with HFMD infection. By using a time series analysis, Huang et al. found that temperature and relative humidity were statistically related to HFMD occurrence [11]. However, a study carried out by Li et al. showed atmospheric pressure having a negative relationship with HFMD incidence in Guangzhou city [12]. Although several spatial analyses on HFMD prevalence have been conducted, only few studies have focused their analyses on determinant factors which attributed to HFMD spatio-temporal clusters [13–15]. Most studies only described the spatial patterns and cluster locations of cases. The objective of our study was to map county-level epidemiological characteristics and spatio-temporal distribution of HFMD incidence and to explore the determinants of HFMD in different clustering areas. Findings of the study were to guide the allocation of public health resources to maximize cost-effectiveness in the prevention and control of HFMD epidemics.

## Methods

### Study area

Hunan province is one of the central inland provinces of China situated between 108°47′ to 114°15′ east longitude and 24°38′ to 30°08′ north latitude with a typically subtropical monsoon climate. Hunan, with a population of approximately 67.8 million, covers an area of about 211,800 km^2^, and has 14 districts and 124 counties. High humidity, abundant heat, high population density and high levels of migration promote the development and spread of enteroviruses within the Province.

### Data description

Surveillance data on HFMD in Hunan was obtained from the China Information System for Disease Control and Prevention (http://www.chinacdc.cn). This data covered the study period (2009 to 2015), and included case types as well as pathogen types (only for laboratory diagnosed cases) for all the new cases identified during the study period. The diagnostic criteria were based on the 2010 HFMD Diagnosis and Treatment Guidelines by the National Health and Family Planning Commission of the People’s Republic of China. Cases without home addresses, clinical or laboratory diagnosis information were excluded from the study. Meteorological data was obtained from the Hunan Meteorological Administration. The Hunan map at county level was also provided by the Information department of the China Disease Control and Prevention Center.

### Spatial autocorrelation analysis

Spatial autocorrelation is defined as the spatial dependence among the given attribute value of one geographic unit and its neighboring units, which lies in almost all spatial observations [16]. Global spatial autocorrelation is used to measure the overall clustering tendency in the study region while the local one can be further used to clarify the patterns and the exact location of the clusters among local counties [17–19]. In this study, we used global Moran’s I statistic and local indicators of spatial autocorrelation (LISA) to investigate spatial association regarding incidence of HFMD in the Hunan province [20].

The global *Moran’s I* statistic ranges from −1 to 1. An *I* > 0 indicates positive autocorrelation and the closer it is to 1, the more aggregated the distribution is. An *I* < 0 indicates negative autocorrelation and the closer it is to −1, the more dispersed the distribution is. An *I* = 0 indicates no autocorrelation, suggesting that the disease is randomly distributed. The significance of Moran’s I is assessed by Monte Carlo test using Z statistic and *P value* (Z ≥ 1.96 or Z ≤ −1.96 indicates statistical significance at the 5% level) [21]. By using the LISA map, four patterns of spatial correlation can be detected: high-high clusters; low-low clusters; high-low clusters; and low-high clusters [22]. In reality, the high-high clusters (i.e. high-incidence districts surrounded by other high-incidence districts) are the most important patterns for the purpose of disease prevention and control.

The formula for global or local *Moran’s I* is:

$$ {Moran}^{\prime }s\ I=\frac{n\sum_{i=1}^n\sum_{j=1}^n{w}_{ij}\left({x}_i-\overline{x}\right)\left({x}_j-\overline{x}\right)}{\left(\sum_{i=1}^n\sum_{j=1}^n{w}_{ij}\right)\sum_{i=1}^n{\left({x}_i-\overline{x}\right)}^2} $$

$$ E(I)=\frac{1}{n-1} $$

$$ Z=\frac{I-E(I)}{\sqrt{Var(I)}} $$

Where n is the number of districts or counties; *x*_*i*_ and *x*_*j*_ are the incidence rates in districts or counties *i* and *j*;$\overline{x}$ is the average incidence rate in the entire study area; and *w*_*ij*_ is the spatial weight between districts or counties *i* and *j*. In this study, by choosing the binary spatial weight matrix in Rook contiguity rule [23], Geoda 1.6 software was used for the spatial autocorrelation analysis whilst ArcGis 10.2 software was used to visualize the results.

### Spatio-temporal cluster analysis

Kulldorff’s method of retrospective space-time scan statistic was used to detect the geographical clusters of HFMD cases at the county level based on a discrete Poisson model [24]. In this approach, a dynamic cylindrical window was used to scan for different time and geographic area to detect all possible clusters. The circular geographic base moves with the radius varying with the population range of the area, while the height varying with the defined time interval [25]. For each scanning, the relative risk (RR) is calculated using the ratio of the observed case number to the expected case number within and outside the windows as well as the log likelihood ratio (*LRR*). The *P* values for clusters detected are calculated by Monte Carlo randomization method [26, 27]. The window with the maximum *LLR* is assumed to be the most likely cluster, and other windows with a statistically significant *LLR* are defined as secondary clusters ranked according to their *LLR* value.

The *LLR* for a given window can be calculated as follows:

$$ LLR=\log {\left(\frac{n}{E(n)}\right)}^n{\left(\frac{N-n}{N-E(n)}\right)}^{N-n}{I}^{\hbox{'}} $$

Where N is the total number of cases; n is the observed number of cases within the scan window; *E*(*n*) and *N* − *E*(*n*) are the expected number of cases within and outside the scan window under the null hypothesis (H_0_: The spatio-temporal clustering of the study area are caused by random factors.), respectively; and *I* ^′^is an indicator function. It is equal to 1 when the window has more cases than expected under the null hypothesis, otherwise it is 0. In space-time scan analysis, the spatial unit is county, with 124 counties in Hunan Province; the temporal unit is month, with 84 months from 2009 to 2015. The spatial size of scanning window was limited to 30% of the total population at risk whilst the temporal size was set to 30% of the total study period in order to scan for small to large clusters. The number of Monte Carlo randomization was set at 999. By setting the time frame of the scan analysis to be one month, we can control the time trends and observe the cluster changes in the entire study. SaTScan 9.4 software was used to perform the spatiotemporal cluster analysis of HFMD and ArcGis 10.2 software was used to visualize the results.

### Autologistic regression model

Spatial autocorrelation is frequently present in the observations and it appears that neighboring units tend to have more similar value than those that are distributed randomly [28]. In considering spatial dependence, Besag et al. present the autologistic regression model which is a special type of ordinary logistic model introducing a spatial autocorrelation term (*SAuto cov*_*i*_) in the form of weighting coefficients [29]. Temporal autocorrelation commonly exists in infectious disease. In our study, we modified the autologistic regression by introducing *TAuto cov*_*i*_ as the temporal autocovariate, which is set with the incidence cases of last month in the same county. Meanwhile, many previous studies have found that population density is significantly associated with HFMD occurrence [30, 31], so we introduced the population density *cov*_*i*_ as the covariate to adjust for population density in different counties.

For the modified autologistic regression model, the form of the model is given by equation:

$$ \mathit{\log}\left(\frac{P_i}{1-{P}_i}\right)={\beta}_0+{\beta}_1{x}_{1,i}+{\beta}_2{x}_{2,i}+\dots +{\beta}_l\ {cov}_i+{\beta}_m SAuto\ {cov}_i+{\beta}_n TAuto\ {cov}_i $$

The probability of the occurrence of the outcome can be illustrated as follows:

$$ {P}_i\left({y}_i=1|{\beta}_0,\beta, p,q,r\right)=\frac{\exp \left({\beta}_0+{\beta}_1{x}_{1,i}+\dots +p\ {cov}_i+ qSAuto\ {cov}_i+ rTAuto\ {cov}_i\right)}{1+\left({\beta}_0+{\beta}_1{x}_{1,i}+\dots +p\ {cov}_i+ qSAuto\ {cov}_i+ rTAuto\ {cov}_i\right)} $$

where *y*_*i*_ is the dependent variable which refers to the probability of spatiotemporal clusters occurrence, *P*_*i*_ is the mean of *y*_*i*_, or equivalently, the probability of *y*_*i*_=1, and *x* denotes the exposure factors in meteorology (the temporal resolution of independent variables is one month which coincide with the time frame of the SaTScan analysis). *cov*_*i*_ is the population density covariate. *SAuto cov*_*i*_, *TAuto cov*_*i*_ are the spatial and temporal autocovariate, respectively. *β*, *p*, *q* and *r* are the regression coefficient. *i* is the index of the county.

$$ SAuto\ {cov}_i=\frac{\sum_{j=1}^{k_i}{w}_{ij}{P}_j}{\sum_{j=1}^{k_i}{w}_{ij}} $$

*SAuto cov*_*i*_ is the weighted average of the probabilities of the geographic unit *i* which is surrounded by other *k*_*i*_ geographic units. *w*_*ij*_ is the spatial weight between county *i* and *j*, $$ {w}_{ij}=\raisebox{1ex}{$1$}\!\left/ \!\raisebox{-1ex}{${h}_{ij}$}\right. $$ and *h*_*ij*_ is the Euclidean distance between the centroids of county *i* and *j*. *P*_*j*_ is the probability of the event occurring in the neighbors of county *i*, which is equal to 1 when the event occurs, otherwise it is 0. In this study, *P*_*j*_ refers to the probability of spatiotemporal clusters occurrence in neighboring county *j* of county *i.* A county with the distance within 31,250 m from county *i* was defined as the neighbors of county *i* [31]. (see Additional file 1: Table S1–1, Table 2).

In this study, after calculating the variance inflation factor (VIF) and tolerance to decrease multicollinearity (see Additional file 1: Table S3–1), five independent variables were selected: monthly average rainfall, monthly average temperature, monthly average wind speed, monthly average relative humidity, monthly total sunshine. We also introduced the population density as the covariate. All variables were standardized. A forward stepwise method was used in the ordinary logistic regression model. We then introduced the *SAuto cov*_*i*_ and *TAuto cov*_*i*_ until each variable was statistically significant. Cox & Snell R^2^ and Nagelkerke R^2^ were used to test the goodness of fit of logistic or autologistic model. The higher the statistic value are, the better the model fits the observations. We used the Relative Operating Characteristic (ROC) to compare the probability predicted by the model with the reality (the occurrence of the spatiotemporal clusters). Closer to 1 means a better fit, which rages from 0.5 to 1. SPSS 22.0 software was used to conduct the autologistc regression model and ArcGIS 10.2 software was used to perform spatial analysis.

## Results

### Epidemiological characteristics

There were 895,429 HFMD cases reported in Hunan Province from 2009 to 2015, with the average annual incidence rate of 194.57 per 100,000 (ranged from 54.31 per 100,000 in 2009 to 318.05 per 100,000 in 2014). Of the total cases, 8263 (0.92%) were severe and 352 (0.39‰) were death.

A total of 559,033 male cases and 336,396 female cases were reported during the study period and the male-to-female incidence ratio was 1.66:1 (ranged from 2.01:1 in 2009 to 1.53:1 in 2014). Most patients were younger than 5 years old, accounting for more than 90% of all reported cases. Scattered children have the highest incidence rate, followed by nursery children and school students. Among 30,377 laboratory confirmed cases, CoxA16, EV71 and other EV accounted for 16.67%, 47.62% and 35.72%, respectively. The predominant pathogen was EV71 in most years except for 2013 and 2015 (Table 1).

**Table 1:** Epidemiological characteristics of HFMD cases in Hunan Province, 2009–2015

	2009	2010	2011	2012	2013	2014	2015	Total
Age								
0–5 year	31,501	102,376	96,131	176,097	102,914	197,243	128,383	834,645
>5 year	3174	9821	6315	13,410	5288	15,549	7227	60,784
Gender								
Male	23,149	71,753	65,700	120,531	66,798	128,533	82,569	559,033
Female	11,526	40,444	36,746	68,976	41,404	84,259	53,041	336,396
Sex ratio	2.01:1	1.77:1	1.79:1	1.75:1	1.61:1	1.53:1	1.56:1	1.66:1
Living condition								
Scattered children	25,379	88,587	86,752	165,220	96,111	182,186	119,264	763,499
Nursery children	7957	19,124	12,847	18,668	9814	25,134	13,728	107,272
School students	1170	4045	2519	5180	2015	4929	2190	22,048
Others	169	441	328	439	262	543	428	2610
Pathogen								
EV71	152	2520	1006	4510	1565	2675	2037	14,465
CoxA16	140	655	1019	685	630	1595	537	5061
Others	112	1186	911	1689	2177	1811	2965	10,851

**Fig. 1:**
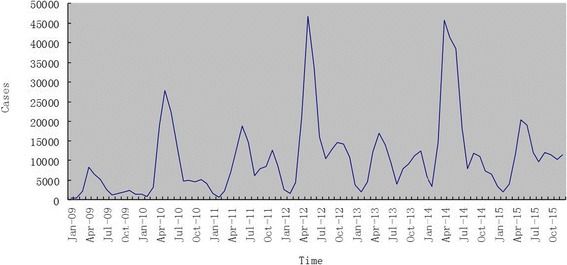
The distribution of HFMD cases in month in Hunan Province, 2009–2015

The monthly distribution of HFMD cases in Hunan is shown in Fig. 1, which indicates that the major peak appeared between April and July whilst the minor peak observed between September and November. The occurrence of HFMD shows significant seasonality with the wave-like increasing tendency.

### Spatial autocorrelation analysis

**Fig. 2:**
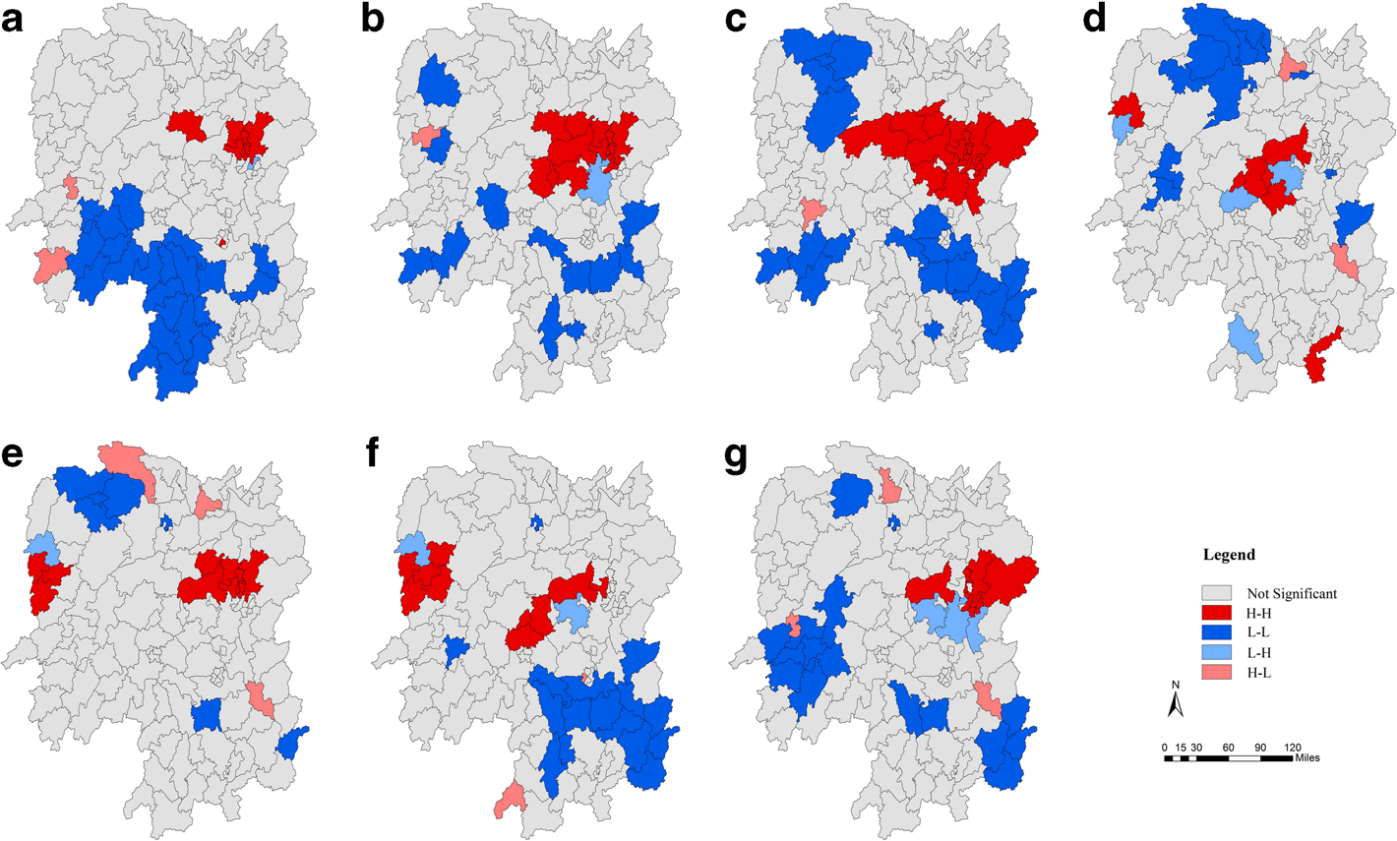
Local Indicators of Spatial Association (LISA) maps for the incidence of HFMD in Hunan Province, 2009–2015 (**a**-**g**); H-H, high-high cluster; L-L, low-low cluster; L-H, low-high cluster; H-L, high-low cluster

Global Moran’s I statistics was performed to investigate the presence of global autocorrelation, which were listed in Table 2. It clearly shows that the HFMD cases were not randomly distributed and that there was high global autocorrelation among HFMD number at county level in Hunan Province. The results of local autocorrelation analysis were mapped in Fig. 2. The LISA map shows that the high-high clusters (dark red color) were mostly in central and northern districts including Changsha, Yiyang, Loudi, Xiangtan, while southwestern districts of Hunan Province were low-low clusters (dark blue color). Nevertheless, contrary to other cities in western districts, Xiangxi showed a high-high pattern in the LISA map in recent years.

**Table 2:** Results of the spatial autocorrelation test on HFMD incidence at county level in Hunan Province, 2009–2015

Year	Moran’ I	S.E.	Z-score	*P*-value
2009	0.374	0.045	8.31	<0.001
2010	0.414	0.043	9.68	<0.001
2011	0.449	0.043	10.40	<0.001
2012	0.299	0.039	7.58	<0.001
2013	0.278	0.043	6.44	<0.001
2014	0.319	0.045	7.04	<0.001
2015	0.362	0.045	8.01	<0.001

### Spatio-temporal cluster analysis

**Fig. 3:**
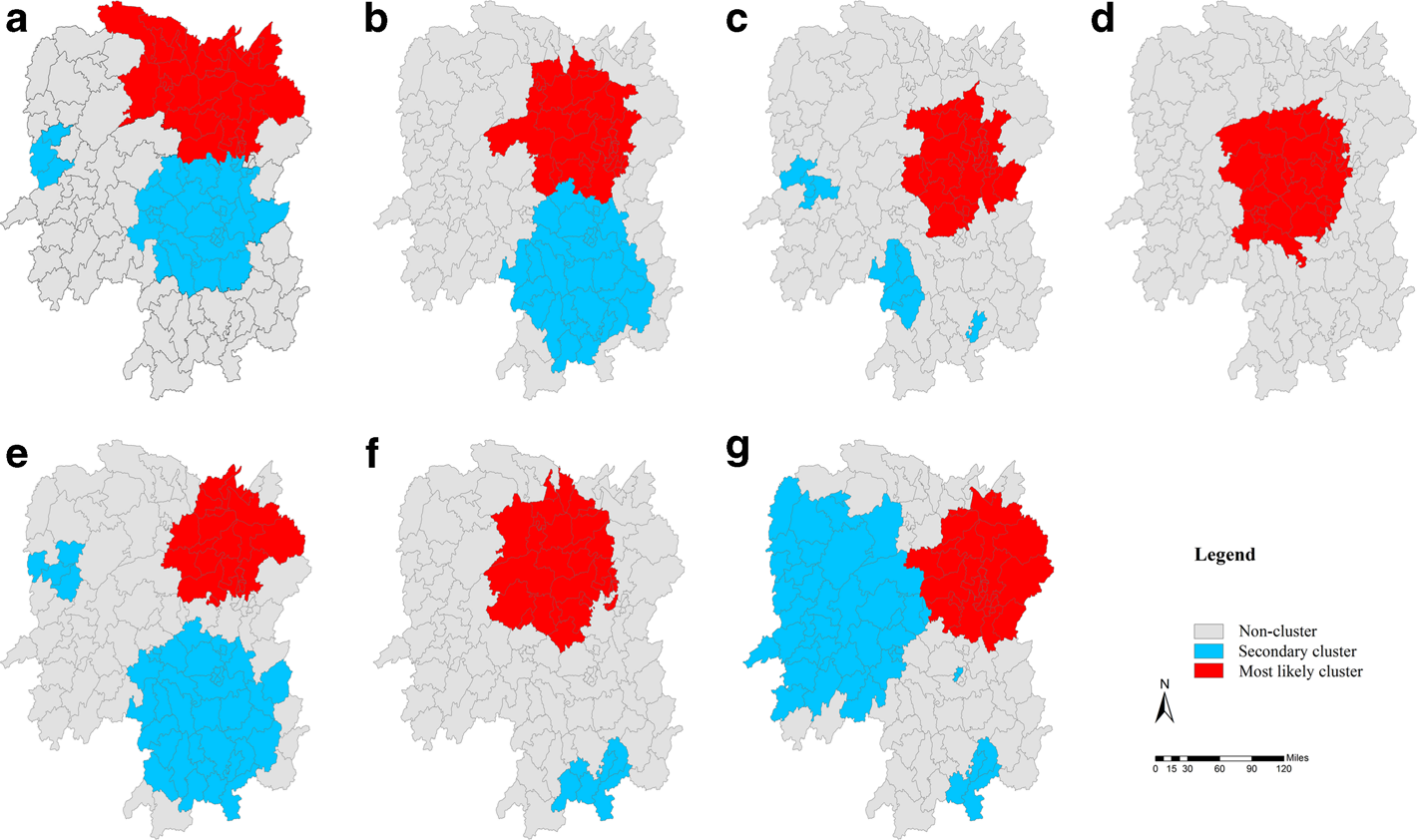
Spatio-temporal clusters of HFMD in Hunan Province, 2009–2015 (**a**-**g**)

Table 3 and Fig. 3 show the scanning results of statistically significant spatial-temporal clusters of HFMD from 2009 to 2015. Seventeen (17) spatial-temporal clusters were detected each year during the study period, including 7 most likely clusters and 10 secondary clusters. All the clusters occurred in the period of the major peak of incidence except for 3 secondary clusters in 2011 and 2015. Clustering areas gathered in the northern regions in 2009, in the central regions from 2010 to 2012, moved to central-southern regions in 2013 and 2014, and central-western regions in 2015.

**Table 3:** Results of the Spatio-temporal cluster test on HFMD incidence at county level in Hunan Province, 2009–2015

Year	Cluster type	Cluster time	Radius (km)/Counties (n)	Cluster regions	RR	LLR	*P*-value
2009	Most-likely	Apr 1-Jun 30	158.08 (32)	Northern	5.33	7631.47	<0.001
	Secondary	Apr 1-May 31	112.49 (33)	Central	2.57	1231.69	<0.001
2010	Most-likely	Apr 1-Jul 31	105.46 (31)	Central	5.48	29,371.10	<0.001
	Secondary	Apr 1-Jun 30	124.80 (30)	Central-southern	2.80	2746.93	<0.001
2011	Most-likely	May 1-Jul 31	96.49 (29)	Central-western	4.27	14,620.54	<0.001
	Secondary	Mar 1-Jul 31	45.44 (4)	Central	2.75	1061.01	<0.001
	Secondary	Sep 1-Dec 31	29.03 (3)	Western	2.29	354.38	<0.001
2012	Most-likely	May 31-Jun 30	111.41 (32)	Central	4.09	20,059.96	<0.001
2013	Most-likely	Apr 1-Jul 31	91.83 (23)	Central-northern	2.54	5221.37	<0.001
	Secondary	Apr 1-Jun 30	39.20 (4)	Western	5.11	1603.22	<0.001
	Secondary	Apr 1-Jun 30	131.84 (36)	South-western	1.72	1409.81	<0.001
2014	Most-likely	Apr 1-Jun 30	117.38 (30)	Central	4.40	33,910.07	<0.001
	Secondary	May 1-Jun 30	74.22 (6)	Southern	2.94	1540.24	<0.001
2015	Most-likely	May 1-Jul 31	120.81 (30)	Central	3.03	9577.40	<0.001
	Secondary	May 1-Oct 30	11.38 (2)	Central	4.04	1314.12	<0.001
	Secondary	May 1-Jun 30	170.72 (38)	Western	1.58	762.17	<0.001
	Secondary	Apr 1-Jun 30	60.65 (4)	Southern	2.38	568.19	<0.001

### Autologistic regression model

Considering the spatial and temporal autocorrelation which existed in Hunan HFMD cases, this study introduced the auto covariates *SAuto cov*_*i*_ and *TAuto cov*_*i*_ on the basis of the ordinary logistic regression model (see Additional file 1: Table S1–4). The occurrence of the HFMD spatio-temporal cluster in the given county was the dependent variable (It is equal to 1 when the spatiotemporal cluster occurs, otherwise it is 0), while the selected exposure factors were the independent variables (they were continuous variables so should not to be assigned). Table 4 shows the results of the autologistic regression. The OR values for monthly average rainfall, monthly average temperature and monthly average relative humidity are all greater than 1, which indicates that these variables are risk factors that are positively related to the occurrence of HFMD spatial-temporal clusters. On the contrary, the OR value for monthly average wind speed is less than 1.

**Table 4:** The OR values obtained from autologistic regression model

Variables	*β*	*S.E.*	*Wald χ* ^*2*^	*OR (95% CI)*	*P-value*
Rainfall (ml)	0.783	0.163	23.053	2.187 (1.587 ~ 3.010)	0.000
Temperature (°C)	1.465	0.319	21.046	4.329 (2.315 ~ 8.096)	0.000
Wind speed (m/s)	−1.356	0.240	31.969	0.258 (0.161 ~ 0.412)	0.000
Humidity (%)	0.727	0.216	11.318	2.070 (1.355 ~ 3.162)	0.001
*Cov*_*i*_	0.470	0.158	8.831	1.600 (1.174 ~ 2.181)	0.003
*SAuto*	6.178	0.497	154.396	482.260 (181.984 ~ 1277.999)	0.000
*TAuto*	0.006	0.001	22.036	1.006 (1.004 ~ 1.009)	0.000
Constant	−5.840	0.436	179.075	0.003	0.000

In addition, the goodness of the model was measured by the Cox & Snell R^2^ and Nagelkerke R^2^ statistics and the ROC value was used to compare the reality with the predicted outcome. All values were listed in Table 5 which indicated that the autologistic regression model had a better goodness of fit than the ordinary one.

**Table 5:** The goodness of fit of the logistic and autologistic regression model

Statistics	Logistic regression model	Autologistic regression model
Cox & Snell R^2^	0.254	0.428
Nagelkerke R^2^	0.475	0.802
ROC	0.913	0.983

## Discussion

In this study, we confirmed that Hunan Province was one of the most serious HFMD epidemic areas in China with the average incidence rate of 194.57 per 100,000, which was much higher than the national average of 139.78 per 100,000 [32]. Furthermore, the incidence rate showed a rapidly increasing trend within the recent 4-year period, suggesting that the prevention and control of HFMD was still a major public health problem in the province. Scattered children under 5 years old accounted for more than 85% of all cases, and the incidence rate was especially high in the 1-year-old group, comparing well with previous reports [33, 34]. Poor immunity as well as a lack of available vaccines made it easier for children to succumb to HFMD viruses. Consistent with Deng’s reports [3], our study found that incidence rate among males was about 1.66 times greater than females, and this could be attributed to the fact that males spent more time engaged in outdoor activities thereby exposing them to pathogens.

Although EV71 and CoxA16 were still predominant in newly occurring HFMD cases around the world [35, 36], our study showed that other EV pathogens ranked first in the recent three-year period. Notably, some other reports have suggested that CoxA6, ECHO30 and CoxA10 were going to be the important causative agents for HFMD [37–39]. This means that surveillance systems should look out for new pathogenic strains alongside the regular EV61 and CoxA16 virus to prevent possible outbreaks associated with other new enteroviruses.

Our study showed significant seasonality in the incidence of HFMD (it peaked in April to July and September to November), similar to other studies conducted in Singapore [40] and Taiwan [41]. The first peak might be the result of the warm temperature, abundant rainfall and high atmospheric pressure during the season. Many children enter kindergarten in September thereby increasing the occurrence of the clustering of cases associated with the second minor peak. Thus, measures such as morning checks, case isolation and school closure should be implemented to reduce incidence and spread of HFMD during the high incidence period.

The global spatial autocorrelation analysis showed that the incidence of HFMD in Hunan during the 2009–2015 period was not randomly distributed at county level. The high-high clusters were aggregated in the provincial capital city, Changsha, and its neighboring areas, whereas some counties in rural areas such as Huaihua, Chenzhou and Yongzhou were low-low clusters in the LISA map. These hot spots were mainly located in areas with highly developed economy, high population density, and good health similar to the observations by Deng et al. [3]. Furthermore, spatiotemporal clusters of HFMD cases appeared annually during the study periods. Nearly all the clusters occurred in April to July, which was the same as the major peak of incidence of HFMD in Hunan. Consistent with most previous researches, the most likely clusters stayed almost the same in the urban areas [3, 42, 43], nevertheless the secondary clusters were diverse during the 2009–2015 period with a shifting trend toward the south and west. According to our study, the occurrence of HFMD spatiotemporal clusters is showing a new trend which needs further research to provide early warning signals for HFMD epidemics.

Although the effect of climatic factors on HFMD incidence has been revealed in many previous researches, there is few supporting studies about the effect of weather conditions on HFMD spatiotemporal clusters. Our autologistic regression analysis results showed that monthly average rainfall (OR = 2.187), monthly average temperature (OR = 4.329) and monthly relative humidity(OR = 2.070) were risk factors for HFMD spatiotemporal clusters, which were in agreement with some HFMD incidence reports [12, 44]. Warm weather, abundant rainfall and high humidity may facilitated the reproduction and transmission of enteroviruses, and facilitated the HFMD clusters. We also found that wind speed (OR = 0.258) has negative correlation with HFMD spatiotemporal clusters, and this compares well with observations by Gui et al. [42]. Wind speed may reduce the concentration of viruses per unit volume and could prevent HFMD virus spread through air and direct contact.

Our research showed that autologistic regression model has a higher goodness of fit than the ordinary one. The value of the constant is the prediction residual error of the regression model. The smaller the value is, the better the interpretation function of the model. By introducing the covariates *SAuto cov*_*i*_ and *TAuto cov*_*i*_, the constant decreased significantly whilst the *p*-value of two covariates were less than 0.001,which indicated the existence of spatial and temporal autocorrelation. Thus, autologistic regression model could reduce the inherent residuals and bias of the model and improve the accuracy in evaluating the effect of determinants in spatio-temporal clustering of HFMD.

There are some limitations in this study. Firstly, the quality of case reports in the surveillance system might vary from area to area due to differences in the availability of medical resources [4]. Some mild cases not going to hospitals also resulted in the underreporting of HFMD cases. Secondly, we chose the county as the least spatial unit which might lose some detailed information. It would have been a good idea to use a more finer areal unit scale such as village or community.

## Conclusions

In summary, this study highlighted that the HFMD incidence from 2009 to 2015 in Hunan Province exhibited dynamic spatiotemporal distribution at county level, with the new shifting trend of clustering areas toward south and west. The autologistic regression model has a higher goodness of fit. After controlling for spatial-temporal heterogeneity, some meteorological parameters, especially rainfall, temperature and humidity might be associated with these clusters. Nevertheless, wind speed was the potential protect factors for the spatiotemporal patterns’ change of HFMD. Our findings provide information for a better understanding of epidemic trends and subsequently the development of more effective HFMD prevention and control strategies.

## Additional file

Additional file 1: Supplementary Table S1–S4. (DOCX 32 kb)

## Abbreviations

CoxA10: Coxsackievirus A10
CoxA16: Coxsackievirus A16
CoxA6: Coxsackievirus A6
ECHO30: Enteric Cytopathic Human Orphan Virus 30
EV71: Enterovirus 71

## References

[1] Hosoya M, Kawasaki Y, Sato M, Honzumi K, Kato A, Hiroshima T, Ishiko H, Suzuki H (2006). Genetic diversity of enterovirus 71 associated with hand, foot and mouth disease epidemics in Japan from 1983 to 2003. Pediatr Infect Dis J.

[2] Weng KF, Chen LL, Huang PN, Shih SR (2010). Neural pathogenesis of enterovirus 71 infection. Microbes and infection / Institut Pasteur.

[3] Deng T, Huang Y, Yu S, Gu J, Huang C, Xiao G, Hao Y. Spatial-temporal clusters and risk factors of hand, foot, and mouth disease at the district level in Guangdong Province. **China** *PloS one*. 2013;8(2):e56943. 10.1371/journal.pone.0056943 PMC3578924 23437278

[4] Hu M, Li Z, Wang J, Jia L, Liao Y, Lai S, Guo Y, Zhao D, Yang W (2012). Determinants of the incidence of hand, foot and mouth disease in China using geographically weighted regression models. PLoS One.

[5] Chumakov M, Voroshilova M, Shindarov L, Lavrova I, Gracheva L, Koroleva G, Vasilenko S, Brodvarova I, Nikolova M, Gyurova S (1979). Enterovirus 71 isolated from cases of epidemic poliomyelitis-like disease in Bulgaria. Arch Virol.

[6] Ishimaru Y, Nakano S, Yamaoka K, Takami S (1980). Outbreaks of hand, foot, and mouth disease by enterovirus 71. High incidence of complication disorders of central nervous system. Arch Dis Child.

[7] McMinn P, Stratov I, Nagarajan L, Davis S (2001). Neurological manifestations of enterovirus 71 infection in children during an outbreak of hand, foot, and mouth disease in Western Australia. Clinical infectious diseases : an official publication of the Infectious Diseases Society of America.

[8] Nguyen NT, Pham HV, Hoang CQ, Nguyen TM, Nguyen LT, Phan HC, Phan LT, Vu LN, Tran Minh NN (2014). Epidemiological and clinical characteristics of children who died from hand, foot and mouth disease in Vietnam, 2011. BMC Infect Dis.

[9] Zhang Y, Zhu Z, Yang W, Ren J, Tan X, Wang Y, Mao N, Xu S, Zhu S, Cui A (2010). An emerging recombinant human enterovirus 71 responsible for the 2008 outbreak of hand foot and mouth disease in Fuyang city of China. Virol J.

[10] Xing W, Liao Q, Viboud C, Zhang J, Sun J, Wu JT, Chang Z, Liu F, Fang VJ, Zheng Y (2014). Hand, foot, and mouth disease in China, 2008-12: an epidemiological study. Lancet Infect Dis.

[11] Huang Y, Deng T, Yu S, Gu J, Huang C, Xiao G, Hao Y (2013). Effect of meteorological variables on the incidence of hand, foot, and mouth disease in children: a time-series analysis in Guangzhou. China BMC infectious diseases.

[12] Li T, Yang Z, Di B, Wang M (2014). Hand-foot-and-mouth disease and weather factors in Guangzhou, southern China. Epidemiol Infect.

[13] Huang JX, Wang JF, Li ZJ, Wang Y, Lai SJ, Yang WZ (2015). Visualized exploratory spatiotemporal analysis of hand-foot-mouth disease in southern China. PLoS One.

[14] Samphutthanon R, Tripathi NK, Ninsawat S, Duboz R (2013). Spatio-temporal distribution and hotspots of hand, foot and mouth disease (HFMD) in northern Thailand. Int J Environ Res Public Health.

[15] Wang J, Cao Z, Zeng DD, Wang Q, Wang X, Qian H (2014). Epidemiological analysis, detection, and comparison of space-time patterns of Beijing hand-foot-mouth disease (2008-2012). PLoS One.

[16] Dolan C, O'Halloran A, Bradley DG, Croke DT, Evans A, O'Brien JK, Dicker P, Shields DC (2005). Genetic stratification of pathogen-response-related and other variants within a homogeneous Caucasian Irish population. European journal of human genetics : EJHG.

[17] Flahaut B, Mouchart M, San Martin E, Thomas I (2003). The local spatial autocorrelation and the kernel method for identifying black zones. A comparative approach. Accid Anal Prev.

[18] Mattsson BJ, Zipkin EF, Gardner B, Blank PJ, Sauer JR, Royle JA (2013). Explaining local-scale species distributions: relative contributions of spatial autocorrelation and landscape heterogeneity for an avian assemblage. PLoS One.

[19] Viladomat J, Mazumder R, McInturff A, McCauley DJ, Hastie T (2014). Assessing the significance of global and local correlations under spatial autocorrelation: a nonparametric approach. Biometrics.

[20] Waldhor T (1996). The spatial autocorrelation coefficient Moran's I under heteroscedasticity. Stat Med.

[21] How Spatial Autocorrelation: Moran's I (Spatial Statistics) Works [http://edndoc.esri.com/arcobjects/9.2/net/shared/geoprocessing/spatial_statistics_tools/how_spatial_autocorrelation_colon_moran_s_i_spatial_statistics_works.htm].

[22] Anselin L (1995). Local indicators of spatial association-LISA. Geogr Anal.

[23] Gu H, Zhang W, Xu H, Li P, Wu L, Guo P, Hao Y, Lu J, Zhang D: [predicating risk area of human infection with avian influenza a (H7N9) virus by using early warning model in China]. Zhonghua liu xing bing xue za zhi =Zhonghua liuxingbingxue zazhi 2015, 36(5):470–475. 26080636

[24] Kulldorff M, Huang L, Pickle L, Duczmal L (2006). An elliptic spatial scan statistic. Stat Med.

[25] Kulldorff M, Feuer EJ, Miller BA, Freedman LS (1997). Breast cancer clusters in the northeast United States: a geographic analysis. Am J Epidemiol.

[26] Kleinman KP, Abrams AM, Kulldorff M, Platt R (2005). A model-adjusted space-time scan statistic with an application to syndromic surveillance. Epidemiol Infect.

[27] Kulldorff M, Heffernan R, Hartman J, Assuncao R, Mostashari F (2005). A space-time permutation scan statistic for disease outbreak detection. PLoS Med.

[28] Anselin LS (2006). I; Kho, Y: GeoDa: A introduction to spatial data analysis. Geogr Anal.

[29] Besag J (1974). Spatial interaction and the statistical analysis of lattice systems. J R Stat Soc Ser B Methodol.

[30] Wang Y, Feng Z, Yang Y, Self S, Gao Y, Longini IM, Wakefield J, Zhang J, Wang L, Chen X (2011). Hand, foot, and mouth disease in China: patterns of spread and transmissibility. Epidemiology.

[31] Bo YC, Song C, Wang JF, Li XW (2014). Using an autologistic regression model to identify spatial risk factors and spatial risk patterns of hand, foot and mouth disease (HFMD) in mainland China. BMC Public Health.

[32] Zhuang ZC, Kou ZQ, Bai YJ, Cong X, Wang LH, Li C, Zhao L, Yu XJ, Wang ZY, Wen HL (2015). Epidemiological research on hand, foot, and mouth disease in mainland China. Viruses.

[33] Liu Y, Wang X, Liu Y, Sun D, Ding S, Zhang B, Du Z, Xue F (2013). Detecting spatial-temporal clusters of HFMD from 2007 to 2011 in Shandong Province. China. PloS one.

[34] Ma E, Chan KC, Cheng P, Wong C, Chuang SK (2010). The enterovirus 71 epidemic in 2008--public health implications for Hong Kong. International journal of infectious diseases : IJID : official publication of the International Society for Infectious Diseases.

[35] Liu W, Wu S, Xiong Y, Li T, Wen Z, Yan M, Qin K, Liu Y, Wu J (2014). Co-circulation and genomic recombination of coxsackievirus A16 and enterovirus 71 during a large outbreak of hand, foot, and mouth disease in Central China. PLoS One.

[36] Tian H, Zhang Y, Sun Q, Zhu S, Li X, Pan Z, Xu W, Xu B (2014). Prevalence of multiple enteroviruses associated with hand, foot, and mouth disease in Shijiazhuang City, Hebei province, China: outbreaks of coxsackieviruses a10 and b3. PLoS One.

[37] Blomqvist S, Klemola P, Kaijalainen S, Paananen A, Simonen ML, Vuorinen T, Roivainen M (2010). Co-circulation of coxsackieviruses A6 and A10 in hand, foot and mouth disease outbreak in Finland. Journal of clinical virology : the official publication of the Pan American Society for Clinical Virology.

[38] Li JL, Yuan J, Yang F, Wu ZQ, Hu YF, Xue Y, Zhou BP, Jin Q (2014). Epidemic characteristics of hand, foot, and mouth disease in southern China, 2013: coxsackievirus A6 has emerged as the predominant causative agent. The Journal of infection.

[39] Mirand A, Henquell C, Archimbaud C, Ughetto S, Antona D, Bailly JL, Peigue-Lafeuille H (2012). Outbreak of hand, foot and mouth disease/herpangina associated with coxsackievirus A6 and A10 infections in 2010, France: a large citywide, prospective observational study. Clinical microbiology and infection : the official publication of the European Society of Clinical Microbiology and Infectious Diseases.

[40] Ang LW, Koh BK, Chan KP, Chua LT, James L, Goh KT (2001). Epidemiology and control of hand, foot and mouth disease in Singapore. Annals of the Academy of Medicine, Singapore 2009.

[41] Hsu CH, Lu CY, Shao PL, Lee PI, Kao CL, Chung MY, Chang LY, Huang LM (2008). Epidemiologic and clinical features of non-polio enteroviral infections in northern Taiwan in. Journal of microbiology, immunology, and infection = Wei mian yu gan ran za zhi 2011.

[42] Gui J, Liu Z, Zhang T, Hua Q, Jiang Z, Chen B, Gu H, Lv H, Dong C (2015). Epidemiological characteristics and spatial-temporal clusters of hand, foot, and mouth disease in Zhejiang Province, China, 2008-2012. PLoS One.

[43] Liao J, Qin Z, Zuo Z, Yu S, Zhang J (2016). Spatial-temporal mapping of hand foot and mouth disease and the long-term effects associated with climate and socio-economic variables in Sichuan Province, China from 2009 to 2013. Sci Total Environ.

[44] Liu W, Ji H, Shan J, Bao J, Sun Y, Li J, Bao C, Tang F, Yang K, Bergquist R, et al. Spatiotemporal dynamics of hand-foot-mouth disease and its relationship with meteorological factors in Jiangsu Province. China. PloS one. 2015;10(6):e0131311. 10.1371/journal.pone.0131311 PMC4488144 26121573

