# Supplementary material for: Spatio-temporal clustering analysis and its determinants of hand, foot and mouth disease in Hunan, China, 2009–2015
Source: BMC Infect Dis. 2017 Sep 25;17:645. doi: 10.1186/s12879-017-2742-9 (PMC5613322; doi:10.1186/s12879-017-2742-9)
Supplement: Additional file 1: — Supplementary Table S1–S4. (DOCX 32 kb) [file 12879_2017_2742_MOESM1_ESM.docx]

1. Threshold setting：

Firstly, we calculated the average nearest neighbor distance (ANND=24554.7m), we rounded off ANND to 25000m. Secondly, we chose 12500m, 18750m, 25000m, 31250m, 37500m (which are the Euclidean distance between the centroids of county i and j, 0.5, 0.75, 1, 1.25, 1.5 times of ANND) as threshold to analyze, respectively. Thirdly, we selected the threshold=31250m in final analysis according to the goodness of fit of model.

Table 1-1.The goodness of fit of autologistic regression model in different threshold

|  | 12500m | 18750m | 25000m | 31250m | 37500m |
| --- | --- | --- | --- | --- | --- |
| Cox & Snell R^2^ | 0.315 | 0.320 | 0.358 | 0.428 | 0.423 |
| Nagelkerke R^2^ | 0.589 | 0.599 | 0.671 | 0.802 | 0.792 |
| ROC | 0.939 | 0.944 | 0.957 | 0.983 | 0.983 |

1. Sensitive analysis:

Table 2-1.The results of autologistic regression with the threshold of 12500m

| Variables | *β* | *S.E.* | *Wald χ^2^* | *OR（95%CI）* | *P-value* |
| --- | --- | --- | --- | --- | --- |
| Rainfall | 0.828 | 0.105 | 61.953 | 2.290（1.863~2.814） | 0.000 |
| Temperature | 2.231 | 0.281 | 63.211 | 9.313（5.373~16.142） | 0.000 |
| Wind speed | -0.978 | 0.187 | 27.218 | 0.376（0.260~0.543） | 0.000 |

Table 2-2. The results of autologistic regression with the threshold of 18750m

| Variables | *β* | *S.E.* | *Wald χ^2^* | *OR（95%CI）* | *P-value* |
| --- | --- | --- | --- | --- | --- |
| Rainfall | 0.818 | 0.107 | 58.617 | 2.266（1.838~2.794） | 0.000 |
| Temperature | 2.122 | 0.284 | 55.788 | 8.345（4.782~14.563） | 0.000 |
| Wind speed | -0.892 | 0.189 | 22.298 | 0.410（0.283~0.593） | 0.000 |

Table 2-3. The results of autologistic regression with the threshold of 25000m

| Variables | *β* | *S.E.* | *Wald χ^2^* | *OR（95%CI）* | *P-value* |
| --- | --- | --- | --- | --- | --- |
| Rainfall | 0.697 | 0.121 | 31.295 | 1.971（1.554~2.500） | 0.000 |
| Temperature | 1.972 | 0.331 | 35.445 | 7.188（3.755~13.760） | 0.000 |
| Wind speed | -0.618 | 0.163 | 14.354 | 0.539（0.392~0.742） | 0.000 |
| Total sunshine | -0.702 | 0.221 | 10.108 | 0.496（0.322~0.764） | 0.001 |
| Humidity | 0.384 | 0.181 | 4.515 | 1.468（1.030~2.092） | 0.034 |

Table 2-4. The results of autologistic regression with the threshold of 31250m

| Variables | *β* | *S.E.* | *Wald χ^2^* | *OR（95%CI）* | *P-value* |
| --- | --- | --- | --- | --- | --- |
| Rainfall | 0.783 | 0.163 | 23.053 | 2.187 (1.587~3.010) | 0.000 |
| Temperature | 1.465 | 0.319 | 21.046 | 4.329 (2.315~8.096) | 0.000 |
| Wind speed | -1.356 | 0.240 | 31.969 | 0.258 (0.161~0.412) | 0.000 |
| Humidity  Covi | 0.727  0.470 | 0.216  0.158 | 11.318  8.831 | 2.070 (1.355~3.162)  1.600 (1.174~2.181) | 0.001  0.003 |

Table 2-5. The results of autologistic regression with the threshold of 37500m

| Variables | *β* | *S.E.* | *Wald χ^2^* | *OR（95%CI）* | *P-value* |
| --- | --- | --- | --- | --- | --- |
| Rainfall | 0.595 | 0.150 | 15.761 | 1.813（1.351~2.431） | 0.000 |
| Temperature | 1.610 | 0.402 | 16.012 | 5.002（2.273~11.004） | 0.000 |
| Wind speed | -0.602 | 0.201 | 8.951 | 0.548（0.369~0.813） | 0.003 |
| Total sunshine  *Cov_i_* | -0.663  0.428 | 0.284  0.144 | 5.450  8.853 | 0.515（0.295~0.899）  1.535（1.157~2.035） | 0.020  0.003 |

According to the OR values obtained from autologistic regression model in different thresholds, we found that the results are relatively stable.

1. Multicollinearity

Multicollinearity is a statistical phenomenon in which two or more covariates in a multiple regression model are highly correlated. Multicollinearity can result in unreasonable equation parameters and cause variables to be non-significant. In this study we used the variance inflation factor (VIF) and tolerance to estimate the multicollinearity, and took 10 as the threshold value of VIF and 0.1 as the threshold value of tolerance. The results of multicollinearity analysis are listed in Table 3-1.

Table 3-1. Results of multicollinearity analysis for meteorological variables

| Exposure factors | Tolerance | VIF |
| --- | --- | --- |
| Rainfall | 0.633 | 1.580 |
| Temperature | 0.280 | 3.571 |
| Air pressure | 0.578 | 1.729 |
| Wind speed | 0.701 | 1.426 |
| Total sunshine | 0.279 | 3.578 |
| Humidity | 0.611 | 1.637 |

1. Logistic regression model

Table 4-1. The results of logistic regression analysis

| Variables | *β* | *S.E.* | *Wald χ^2^* | *OR（95%CI）* | *P-value* |
| --- | --- | --- | --- | --- | --- |
| Rainfall | 0.727 | 0.098 | 54.624 | 2.069（1.706~2.509） | 0.000 |
| Temperature | 2.869 | 0.284 | 101.833 | 17.628(10.096~30.778） | 0.000 |
| Wind speed | -0.947 | 0.172 | 30.375 | 0.388（0.277~0.543） | 0.000 |
| Humidity | 0.333 | 0.140 | 5.649 | 1.395（1.060~1.836） | 0.017 |
| *Cov_i_* | 0.454 | 0.088 | 26.695 | 1.574（1.325~1.869） | 0.000 |
| Constant | -3.817 | 0.254 | 225.595 | 0.022 | 0.000 |
